# Empirical progression criteria thresholds for feasibility outcomes in HIV clinical trials: a methodological study

**DOI:** 10.1186/s40814-023-01342-x

**Published:** 2023-06-14

**Authors:** Lawrence Mbuagbaw, Lucy Huizhu Chen, Eunice Aluko, Maya Stevens-Uninsky, Akudo C. J. Eze-Onuorah, Michael Cristian Garcia, Larysa Stech, Tariq Atkin-Jones, Nadia Rehman, Amidu Raifu

**Affiliations:** 1 Department of Health Research Methods, Evidence, and Impact, McMaster University, 1280 Main Street West, Hamilton, ON L8S4L8 Canada; 2 Centre for Development of Best Practices in Health (CDBPH), Yaoundé Central Hospital, Yaoundé, Cameroon; 3 Department of Medicine, McMaster University, Hamilton, ON Canada; 4 Department of Anesthesia, McMaster University, Hamilton, ON Canada; 5 Department of Pediatrics, McMaster University, Hamilton, ON Canada; 6 Biostatistics Unit, Father Sean O’Sullivan Research Centre, St Joseph’s Healthcare Hamilton, Hamilton, ON Canada; 7 Division of Epidemiology and Biostatistics, Department of Global Health, Stellenbosch University, Cape Town, South Africa; 8 Faculty of Arts and Science, University of Toronto, Toronto, ON Canada; 9 Department of Global Health, Faculty of Health Sciences, McMaster University, Hamilton, ON Canada; 10 Faculty of Health Sciences, McMaster University, Hamilton, ON Canada; 11 Temerty Faculty of Medicine, University of Toronto, Toronto, Canada; 12 Public Health, Faculty of Applied Sciences, Brock University, St. Catharines, ON Canada

**Keywords:** HIV, Pilot trial, Feasibility trial, Progression criteria, Threshold, Clinical trials

## Abstract

**Introduction:**

Pilot and feasibility trials use predetermined thresholds for feasibility outcomes to decide if a larger trial is feasible. These thresholds may be derived from the literature, observational data, or clinical experience. The aim of this study was to determine empirical estimates for feasibility outcomes to inform future HIV pilot randomized trials.

**Methods:**

We conducted a methodological study of HIV clinical trials indexed in the past 5 years (2017–2021) in the PubMed database. We included trials of people living with HIV individually randomized to any type of intervention and excluded pilot trials and cluster randomized trials. Screening and data extraction were conducted in duplicate. We computed estimates for recruitment, randomization, non-compliance, lost to follow-up, discontinuation, and the proportion analyzed using a random effects meta-analysis of proportions and reported these estimates according to the following subgroups: use of medication, intervention type, trial design, income level, WHO region, participant type, comorbidities, and source of funding. We report estimates with 95% confidence intervals.

**Results:**

We identified 2122 studies in our search, of which 701 full texts were deemed relevant, but only 394 met our inclusion criteria. We found the following estimates: recruitment (64.1%; 95% CI 57.7 to 70.3; 156 trials); randomization (97.1%; 95% CI 95.8 to 98.3; 187 trials); non-compliance (3.8%; 95% CI 2.8 to 4.9; 216 trials); lost to follow-up (5.8%; 95% CI 4.9 to 6.8; 251 trials); discontinuation (6.5%; 95% CI 5.5 to 7.5; 215 trials); analyzed (94.2%; 95% CI 92.9 to 95.3; 367 trials). There were differences in estimates across most subgroups.

**Conclusion:**

These estimates may be used to inform the design of HIV pilot randomized trials with careful consideration of variations due to some of the subgroups investigated.

## Key messages regarding feasibility

- 1) What uncertainties existed regarding the feasibility?
  Not applicable
- 2) What are the key feasibility findings?
  Not applicable
- 3) What are the implications of the feasibility findings for the design of the main study?
  Not applicable

## Introduction

Preliminary studies are commonly used to inform the design of clinical trials. In the past decade there has been an increasing emphasis on the importance of conducting preliminary trials prior to a definitive large-scale trial in order to increase efficiency and reduce research waste [1]. These studies are often called “pilot studies” or “feasibility studies” and have been found to be very effective in reducing research waste such as over-spending [2]. Although the terms “pilot studies” and “feasibility studies” are used interchangeably, there are some key differences. A study or trial can be labeled as “pilot” when it is a small-scale study conducted prior to the large-scale study, mimicking the design of the main study, and designed to test and refine a protocol (i.e., ensure recruitment protocols are efficient, provide training and experience in running randomization, treatments, and follow-up assessments). In contrast, feasibility studies are designed to evaluate whether a larger scale study could be performed and used to estimate important parameters required to design the main study (i.e., willingness of participants to be randomized, number of people eligible, response rates, follow-up rates, etc.) [3, 4].

Pilot and feasibility studies may use progression criteria to determine if a larger study is feasible. Progression criteria are one or more feasibility outcomes that must meet a pre-defined threshold for feasibility to be declared. They inform the decision to move forward to a larger trial, make modifications to the larger trial, or abandon altogether [5]. For example, investigators could determine that a larger trial is feasible if they are able to recruit 75% of the people that are approached. Progression criteria are insufficiently used in pilot studies [6, 7], despite the requirement to declare progression criteria in the CONSORT extension for pilot randomized controlled trials (RCTs) [3]. This creates challenges with how pilot studies are interpreted and how decisions are made with regards to a larger trial.

Pilot studies are particularly useful in HIV research due to the numerous challenges with recruiting and retaining participants, who may be experiencing social stigma and discrimination. Moreover, people living with HIV (PLWH) may belong to other minority groups associated with discrimination (i.e.; Black people, people who inject drugs [PWID] and men who have sex with men [MSM]) [2]. Considering the over-representation of intersectional discrimination in HIV studies, pilot studies would provide an invaluable service in determining potential recruitment challenges in these specific population groups. In a sample of 248 pilot studies in HIV research, the authors noted that pilot studies are increasingly being used [2]. However, several design, analysis, and reporting issues exist including limited use of progression criteria and lacking justifications for trial sample sizes [2].

Researchers may face challenges in defining feasibility outcomes and developing progression criteria due to the lack of empirical data on credible and reasonable thresholds for frequently used outcomes such as recruitment, compliance, and dropouts. There is limited guidance on how to set these thresholds. In a methodological study, only 28% of publications provided a rationale for their progression criteria [7]. Existing guidance cites the use of prevalence or incidence rates and pre-existing observational data for recruitment rates [8]. However, observational data may not always be available and, even if they are, they may not necessarily reflect estimates that would be true for a randomized trial. A potential solution to this issue is to summarize the estimates from completed full scale trials.

The purpose of this study is to inform the design of HIV clinical trials by providing credible evidence-based estimates to use in determining progression criteria thresholds when planning feasibility outcomes in HIV randomized clinical trials.

## Methods

### Data collection

We conducted a methodological study of HIV clinical trials indexed in the past 5 years (2017–2021) in the PubMed database using the following search strategy (LM):

- ((((randomized controlled trial [pt]) OR (controlled clinical trial [pt]) OR (randomized [tiab]) OR (placebo [tiab]) OR (clinical trials as topic [mesh: noexp]) OR (randomly [tiab]) OR (trial [ti])) NOT (animals [mh] NOT humans [mh])) AND ((HIV) OR (human-immunodeficiency-virus) OR (human immunodeficiency virus)) NOT ((pilot [ti]) OR (feasibility [ti]) OR (protocol [ti])))

The results of our search were collected in EndNote reference manager. Reviewers working independently screened all the titles and abstracts for eligibility (LC, EA, MSU, ACJE, MCG, LS, TAJ, NR). To be eligible, a trial must include only people living with HIV individually randomized to any type of intervention. We excluded pilot or feasibility RCTs, trials with cluster randomization, trials in which participants were enrolled as couples (dyads) and trials published only as abstracts.

### Data extraction

Full text articles were retrieved for potentially eligible articles and screened in duplicate. Data were extracted by one reviewer and verified by a second independent reviewer for quality control (LC, EA, MSU, ACJE, MCG, LS, TAJ, NR). The following data were extracted: basic bibliometric information (author name, author contact information, year of publication, and journal), country of origin, country’s income level (based on the World Bank Classification as high, upper middle, lower middle and low) [9], World Health Organization (WHO) region (Africa, Americas, Eastern Mediterranean, Europe, South East Asia, Western Pacific) [10], source of funding (industry, non-industry), trial duration in months, trial design (crossover, multi arm, factorial), follow-up duration, number of trial sites, use of medication (pharmacological versus non pharmacological), intervention type (Educational, Mobile health, Counselling, Electronic, Change in healthcare delivery, Incentives, Peer support, Psychotherapy, Outreach), population type known to be at higher risk of HIV infection and morbidity (Black people, MSM, women, youth, PWID, people in prisons, transgender people and children) [11, 12], comorbidities (tuberculosis, mental health, substance use, cancer). The following metrics were extracted from the CONSORT flow diagram, tables, or manuscript text: number of participants who were assessed for eligibility, recruited, randomized, who did not receive the intervention as planned, lost to follow-up, who discontinued intervention, and the number analyzed. Data extraction was conducted using DistillerSR (Evidence Partners, Ottawa, Canada).

### Data analysis

#### We computed the following metrics as percentages:

- Recruitment: number enrolled divided by the number approached or assessed
- Randomization: number randomized divided by the number enrolled
- Non-compliance: number who did not receive the intervention as planned divided by the number randomized
- Lost to follow-up: number lost to follow-up divided by the number randomized
- Discontinuation: number who discontinued the intervention divided by the number randomized
- Proportion analyzed: number analyzed divided by the number randomized

The analysis was performed in StataCorp. 2021. *Stata Statistical Software: Release 17*. College Station, TX: StataCorp LLC. These proportions were pooled using random effects models. We used the Freeman-Tukey double arcsine transformation to stabilize the variances. The weighted pooled estimates were then back transformed, and using these transformed values and their variances, the pooled estimates were computed using the inverse variance method. Based on the binomial distribution, the exact 95% confidence intervals (CI) were calculated using the Clopper-Pearson approach. We conducted subgroup analyses based on the use of medication, intervention type, study design, country income level, WHO region, participant type, participant co-morbidities, and source of funding. These data are meant to be descriptive and therefore no interaction analyses were conducted. We also conducted a sensitivity analysis for the studies that reported on all the metrics. The number of studies, pooled estimates, and 95% confidence intervals (CI) are reported. Inferences for subgroups are made only when there are at least two studies.

## Results

**Fig. 1:**
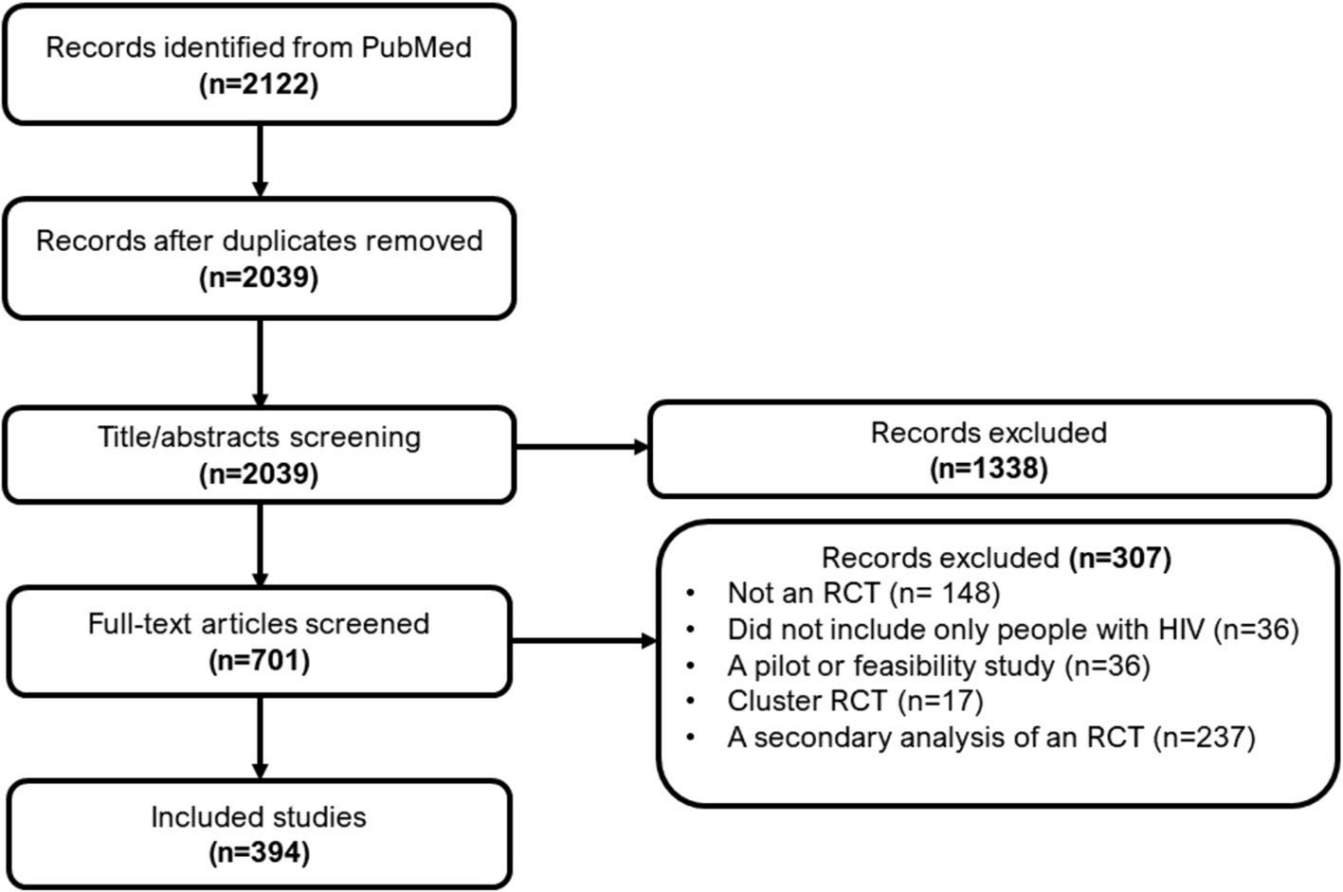
Flow diagram of study selection

Our search retrieved 2122 articles of which 83 were duplicates. Of these articles, 701 were deemed relevant after title and abstract screening. After full text screening, we included 394 articles. The flow of study selection is shown in Fig. 1.

About half of the included trials were of pharmaceutical interventions (212; 53.8%). The largest group of trials involved changes in healthcare delivery, such as changes in the number of pills, home-based care, and task-shifting (182; 46.2%). Seventy-nine (20.1%) were multi-arm trials. The majority were conducted in high income countries (164; 42.6%) and in the Africa region (127; 32.2%). The largest group of people studied were women (65; 16.5%) followed by Black people (39; 10.0%). The most common comorbidity studied was substance use (34; 8.6%). Most trials were non-industry funded (300; 76.1%). Two thirds (66.3%) were multicenter trials with a median number of sites of 3 (quartile 1: quartile 3; 1:7). The mean (standard deviation) duration of follow-up was 11.7 (9.2) months.

These results are summarized in Table 1.

**Table 1:** Characteristics of included studies

Characteristic	Data
**Use of medication: *n* (%)**	
Pharmacological	212 (53.8)
Non-pharmacological	182 (46.2)
**Intervention type: *n* (%)**	
Educational	40 (10.2)
Mobile health	55 (14.0)
Counseling	96 (24.4)
Electronic	15 (3.8)
Change in healthcare delivery	182 (46.2)
Incentives	17 (4.3)
Peer support	13 (3.3)
Psychotherapy	40 (10.2)
Outreach	23 (5.8)
**Type of trial: *n* (%)**	
Factorial	15 (3.8)
Multi-arm	79 (20.1)
Crossover	23 (5.8)
**Income level: *n* (%)**	
High	164 (42.6)
Upper middle	62 (15.7)
Lower middle	65 (16.5)
Low	34 (8.6)
Mixed	69 (17.5)
**WHO region: *n* (%)**	
Africa	127 (32.2)
Americas	124 (31.5)
Eastern Mediterranean	6 (1.5)
Europe	43 (11.9)
South East Asia	17 (4.3)
Western Pacific	14 (3.6)
Mixed	63 (16.0)
**Participant type: *n* (%)**	
Black people	39 (10.0)
Men who have sex with men	21 (5.3)
Women	65 (16.5)
Youth	24 (6.1)
People who inject drugs	25 (6.3)
Prisoners	9 (2.3)
Transgender people	2 (0.5)
Children	18 (4.6)
**Participant comorbidities: *n* (%)**	
TB	22 (5.6)
Mental health	14 (3.6)
Substance use	34 (8.6)
Cancer	6 (1.5)
**Source of funding: *n* (%)**	
Industry alone	45 (11.4)
Non-industry	300 (76.1)
Both	20 (7.4)
**Trial sites**	
Single center	125 (31.7)
Multi-center	269 (66.3)
Trial sites: median (Q1; Q3)*	3 (1;7)
**Duration of follow-up (months): mean (SD)**	11.2 (9.7)

^*^Q1: quartile 1; Q3: quartile 3

### Recruitment

One hundred and fifty-six studies (156) had sufficient data to compute recruitment. The overall recruitment rate was 64.1% (95% CI 57.7 to 70.3). The lowest recruitment rate was in the trials of participants with mental health comorbidities (42.9; 95% CI 22.9 to 64.3; 8 trials) and the highest in trials conducted in more than one WHO region (80.2% 95% CI 73.1 to 86.4; 20 trials).

### Randomization

One hundred and eighty-seven studies (187) had sufficient data to compute randomization. The overall randomization rate was 97.1 (95% CI 95.8 to 98.3). The lowest randomization rate was observed in the trials that used incentives as the intervention (86.8; 95% CI 54.5 to 100.0; 8 trials), and the highest was in the trials conducted in more than one WHO region (99.9; 95% CI 99.7 to 100.0; 27 trials).

### Non-compliance

Two-hundred and sixteen studies (216) had sufficient data to compute non-compliance. The overall non-compliance was 3.8% (95% CI 2.8 to 4.9). The lowest non-compliance was in factorial trials (0.5; 95% CI 0.0 to 1.6; 7 trials), and the highest non-compliance was in trials with a psychotherapy intervention (16.1%; 95% CI 5.9 to 30.0; 16 trials).

### Lost to follow-up

Two hundred and fifty-one studies (*n* = 251) had sufficient data to compute lost to follow-up. The overall lost to follow-up was 5.8% (95% CI 4.9 to 6.8). The lowest lost to follow-up was in the trials conducted with industry funding (1.8%, 95% CI 1.1 to 2.7; 34 trials), and the highest lost to follow-up was in the trials with an educational intervention (15.0%, 95% CI 10.9 to 19.6; 29 trials).

### Discontinuation

Two hundred and fifteen (215) trials had sufficient data to compute discontinuation. The overall discontinuation was 6.5% (95% CI 5.5 to 7.5). The lowest discontinuation was in the trials conducted in South East Asia region (0.6%, 95% CI 0.0 to 2.5; 8 trials), and the highest discontinuation was in the trials with patients who had cancer (16.1; 95% CI 13.2 to 19.2; 2 trials).

### Analyzed

Three hundred and sixty-seven (367) trials had sufficient data to estimate the proportion analyzed. The overall proportion analyzed was 94.2% (95% CI 92.9 to 95.3). The lowest proportion analyzed was in the studies with an electronic intervention (89.0; 95% CI 81.9 to 94.6; 15 trials), and the highest proportion analyzed was in studies with transgender people (99.6; 95% CI 98.8 to 100.0; 2 trials).

All the results are summarized in Table 2.

**Table 2:** Summary of estimates for feasibility outcomes

Subgroup	Recruitment	Randomization	Intervention not received as planned	Lost to follow-up	Discontinued	Analyzed
	***n*; % (95% CI)**	***n*; % (95% CI)**	***n*; % (95% CI)**	***n*; % (95% CI)**	***n*; % (95% CI)**	***n*; % (95% CI)**
**Use of medication**						
**Pharmacological**	72; 69.5 (61.6, 76.9)	89; 98.3 (97.0, 99)	121; 2.7 (1.7, 4.0)	134; 3.7 (2.8, 4.6)	126; 7.9 (6.6, 9.3)	198; 94.9 (93.2, 96.4)
**Non-pharmacological**	84; 59.3 (50.9, 67.4)	99; 95.8 (93.0, 98.0)	95; 5.3 (3.6, 7.4)	117; 8.8 (7.1, 10.7)	89; 4.6 (3.2, 6.3)	172; 93.3 (91.5, 94.8)
**Type of intervention**						
**Educational**	14; 55.4 (42.6, 67.8)	15; 94.4 (79.1, 100.0)	20; 6.0 (1.4, 13.4)	29; 15.0 (10.9, 19.6)	20; 7.4 (3.5, 12.5)	38; 92.5 (88.4, 95.8)
**Mobile health**	28; 63.9 (52.3, 74.6)	31; 93.2 (85.0, 98.4)	27; 3.7 (1.6, 6.7)	34; 8.7 (5.5, 12.5)	26; 7.4 (3.9, 11.9)	52; 93.4 (90.1, 96.2)
**Counseling**	41; 57.6 (48.1, 66.8)	51; 95.8 (91.4, 98.8)	47; 5.3 (2.3, 9.3)	58; 10.2 (7.6, 13.2)	43; 5.3 (3.1, 8.0)	92; 94.9 (92.7, 96.7)
**Electronic**	8; 72.8 (53.8, 88.2)	8; 97.3 (90.5, 100.0)	7; 7.9 (1.4, 18.4)	11; 11.0 (4.4, 20.1)	9; 6.4 (2.1, 12.6)	15; 89.0 (81.9, 94.6)
**Change in healthcare delivery**	81; 67.2 (60.2, 73.8)	94; 98.4 (97.3, 99.2)	105; 2.5 (1.5, 3.7)	119; 3.9 (3.0, 5.0)	115; 7.3 (6.0, 8.6)	174; 95.9 (94.3, 97.3)
**Incentives**	8; 47.1 (22.6, 72.5)	8; 86.8 (54.5, 100.0)	8; 1.4 (0.0, 5.5)	9; 8.2 (1.4, 19.5)	9; 6.1 (1.9, 12.3)	14; 99.4 (96.9, 100.0)
**Peer support**	6; 83.3 (70.5, 93.0)	7; 99.9 (99.4, 100.0)	6; 10.9 (0.5, 31.4)	9; 11.5 (6.1, 18.3)	3; 3.2 (0.7, 7.2)	13; 95.6 (91.0, 98.7)
**Psychotherapy**	23; 51.4 (39.9, 62.8)	30; 95.2 (87.5, 99.5)	16; 16.1 (5.9, 30.0)	18; 9.4 (6.1, 13.2)	18; 5.9 (3.1, 9.5)	37; 96.1 (93.5, 98.2)
**Outreach**	11; 72.0 (45.0, 92.4)	14; 98.7 (96.6, 99.9)	9; 11.4 (4.8, 20.3)	11; 9.0 (3.9, 15.9)	15; 8.5 (4.9, 13.0)	22; 94.8 (88.7, 98.7)
**Type of study**						
**Factorial**	6; 74.9 (49.8, 93.3)	8; 98.3 (91.2, 100.0)	7; 0.5 (0.0, 1.6)	8; 3.6 (1.5, 6.0)	7; 8.4 (3.8, 14.4)	13; 95.7 (89.3, 99.4)
**Multi-arm**	41; 66.5 (52.3,79.4)	48; 98.1 (94.7, 99.9)	43; 4.1 (2.5, 6.0)	49; 7.5 (5.4, 9.9)	41; 5.9 (3.7, 8.6)	75; 95.0 (92.8, 96.9)
**Crossover**	10; 55.7 (38.6, 72.1)	15; 98.5 (95.7, 100.0)	15; 2.8 (0.3, 7.0)	15; 4.2 (1.7, 7.5)	14; 4.2 (1.2, 8.6)	22; 96.1 (92.5, 98.6)
**Income level**						
**High**	54; 60.7 (52.9, 68.2)	20; 96.4 (92.7, 99.0)	89: 4.1 (2.5, 6.1)	102; 7.2 (5.5, 9.0)	91; 7.2 (5.7, 8.8)	153; 93.8 (92.0, 95.4)
**Upper middle**	25; 64.1 (51.4, 75.8)	29; 92.9 (85.9, 97.8)	35; 3.4 (1.4, 5.9)	39; 5.6 (2.8, 9.1)	36; 5.2 (2.7, 8.3)	59; 94.5 (91.3, 97.0)
**Lower middle**	33; 66.7 (50.0, 81.6)	37; 98.0 (95.3, 99.6)	35; 4.3 (1.9, 7.5)	42; 7.5 (5.3, 9.9)	30; 4.9 (3.1, 7.1)	63; 95.3 (93.0, 97.1)
**Low**	17; 52.7 (38.5, 66.7)	20; 96.8 (92.1, 99.6)	17; 5.1 (2.1, 9.3)	25; 6.1 (3.7, 9.2)	17; 2.6 (0.3, 6.8)	31; 92.8 (88.3, 96.2)
**Mixed**	27; 73.9 (55.4, 88.8)	33; 99.6 (99.2, 99.9)	40; 2.6 (1.0, 4.7)	43; 2.3 (1.3, 3.5)	41; 9.5 (7.2, 12.1)	64; 94.3 (90.6, 97.2)
**WHO region**						
**Africa**	56; 62.4 (50.7, 73.4)	66; 98.2 (96.6, 99.3)	68; 4.3 (2.5, 6.4)	87; 5.9 (4.6, 7.4)	66; 4.7 (3.1, 6.5)	122; 94.5 (92.1, 96.4)
**Americas**	47; 54.3 (46.3, 62.2)	58; 94.7 (89.7, 98.2)	68; 5.1 (2.8, 8.0)	78; 8.7 (6.6, 11.1)	70; 6.7 (4.8, 8.8)	117; 93.0 (90.6, 95.1)
**Eastern Mediterranean**	2; 56.6 (50.3, 62.8)	2; 97.9 (94.5, 99.8)	4; 5.8 (2.8, 9.8)	4; 10.4 (0.6, 27.7)	3; 16.3 (1.1,42.0)	6; 89.1 (70.9, 99.3)
**Europe**	14; 78.1 (64.8, 88.9)	16; 96.5 (89.8, 99.9)	29; 2.7 (1.1, 4.8)	28; 3.8 (1.7, 6.4)	25; 8.1 (5.4, 11.2)	43; 95.3 (92.3, 97.5)
**South East Asia**	9; 70.6 (42.5, 92.1)	10; 93.3 (73.7, 100.0)	8; 1.6 (0.0, 5.9)	10; 3.7 (0.1, 10.9)	8; 0.6 (0.0, 2.5)	16; 98.2 (93.4, 100.0)
**Western Pacific**	8; 56.4 (35.5, 76.2)	8; 90.0 (70.7, 99.6)	8; 5.6 (1.0, 13.3)	7; 4.5 (0.8, 10.6)	8; 6.1 (1.6, 13.1)	12; 92.5 (84.7, 97.8)
**Mixed**	20; 80.2 (73.1, 86.4)	27; 99.9 (99.7, 100.0)	31; 1.6 (0.5, 3.1)	37; 2.9 (1.5, 4.7)	35; 10.0 (7.4, 13.0)	54; 94.4 (91.6, 96.6)
**Participant type**						
**Black**	22; 64.7 (46.7, 80.8)	25; 99.8 (99.4, 100.0)	19; 3.1 (0.7, 7.1)	27; 5.4 (3.4, 7.8)	20; 10.2 (6.7, 14.4)	38; 94.3 (88.5, 98.2)
**Men who have sex with men**	12; 50.1 (36.0, 64.2)	15; 95.0 (79.9, 100.0)	11; 7.5 (2.3, 15.2)	14; 10.9 (5.7, 17.5)	6; 10.0 (4.5, 17.4)	19; 94.6 (88.9, 98.3)
**Women**	18; 54.3 (40.1, 68.2)	28; 97.2 (93.6, 99.4)	30; 5.5 (2.6, 9.3)	37; 6.8 (4.1, 9.9)	32; 4.9 (2.9, 7.2)	58; 93.1 (87.7, 97.0)
**Youth**	12; 48.3 (19.6, 77.6)	15; 98.2 (95.3, 99.8)	16; 4.5 (1.6, 8.6)	15; 7.5 (3.6, 12.5)	10; 10.0 (5.7, 15.3)	23; 92.8 (87.8, 96.6)
**People who inject dugs**	13; 43.9 (32.1, 56.0)	13; 91.8 (80.1, 98.7)	17; 2.4 (0.8, 4.8)	15; 14.9 (9.6, 21.1)	13; 6.8 (2.3, 13.3)	24; 97.4 (94.6, 99.2)
**Prisoners**	3; 59.7 (45.8, 72.9)	3; 95.3 (69.1, 100.0)	5; 13.9 (0.0, 56.1)	5; 14.5 (4.2, 29.6)	6; 8.0 (1.1, 19.7)	9; 97.5 (91.9, 100.0)
**Transgender people**	1; 11.8 (10.5, 13.3)	1; 84.4 (79.4, 88.3)	1; 2.3 (1.0, 5.3)	1; 29.8 (25.3, 34.7)	na	2; 99.6 (98.8, 100.0)
**Children**	9; 52.4 (18.0, 85.5)	11; 97.1 (92.0, 99.8)	7; 2.5 (0.3, 6.5)	11; 5.2 (2.3, 9.0)	14; 9.5 (5.9, 13.8)	16; 95.9 (91.1, 99.0)
**Participant comorbidities**						
**Tuberculosis**	7; 64.4 (57.7, 70.03)	7; 98.3 (90.2, 100.0)	15; 2.7 (0.3, 7.0)	17; 7.3 (3.6, 12.0)	4; 8.9 (5.5, 26.8)	21; 94.7 (89.6, 98.2)
**Mental health**	8; 42.9 (22.9, 64.3)	10; 96.4 (86.7, 100.0)	2; 3.1 (1.7, 4.9)	7; 12.5 (5.0, 21.7)	4; 8.9 (0.3, 26.8)	12; 97.8 (94.2, 99.8)
**Substance use**	16; 49.3 (37.3, 61.4)	18; 93.2 (83.5, 99.0)	21; 2.9 (0.8, 5.9)	20; 12.8 (8.7, 17.5)	18; 5.9 (2.4, 10.5)	33; 97.0 (94.2, 99.0)
**Cancer**	6; 66.6 (305, 94.1)	6; 93.0 (69.3, 100.0)	3; 0.6 (0.0,2.3)	4; 11.3 (4.2, 21.0)	2; 16.1 (13.2, 19.2)	6; 95.2 (83.7, 100.0)
**Source of funding**						
**Industry alone**	18; 76.7 (49.3, 95.5)	19; 94.2 (76.8, 100.0)	28; 1.2 (0.4, 2.5)	34; 1.8 (1.1, 2.7)	32; 7.2 (5.9, 8.7)	44; 95.9 (94.1, 97.5)
**Non-industry**	128; 62.7 (55.9, 69.3)	154; 97.2 (95.6, 98.4)	164; 4.2 (3.0, 5.6)	185; 6.7 (5.6, 7.9)	163; 6.6 (5.4, 8.0)	281; 94.0 (92.5, 95.4)
**Both**	4; 57.2 (26.6, 85.0)	5; 94.2 (76.8, 100.0)	9; 5.1 (1.5, 10.4)	12; 4.1 (1.8, 7.2)	9; 6.4 (1.6, 13.8)	20; 90.8 (80.2, 97.7)
**Total**	**156; 64.1 (57.7, 70.3)**	**187; 97.1(95.8–98.3)**	**216; 3.8 (2.8–4.9)**	**251; 5.8 (4.9–6.8)**	**215; 6.5 (5.5–7.5)**	**367; 94.2 (92.9–95.3)**

In our sensitivity analyses, 62 studies reported data on all the outcomes with the following estimates for recruitment (66.9%; 95% CI 58.5 to 74.8), randomization (97.3%; 95% CI 95.1 to 98.9), non-compliance (3.2%; 95% CI 1.3 to 5.6), lost to follow-up (4.9%; 95% CI 3.3 to 6.7), discontinuation (5.0%; 95% CI 3.4 to 6.9), and proportion analyzed (95.8%; 95% CI 93.7 to 97.5).

## Discussion

In this methodological study, we have provided empirical data to use in determining progression criteria thresholds when planning feasibility outcomes in HIV pilot randomized trials. We have also demonstrated that these estimates may vary based on the use of medication in the trials, the type of intervention, study design, income level of the countries in which the trial is conducted, region of the world, type of participants included, the comorbidities they may have, and the source of funding.

This is the first study of its kind to provide estimates intended to inform the design of pilot and feasibility trials in HIV. The estimates and their confidence intervals can be used for sample size calculations for feasibility outcomes and to set thresholds for feasibility. For example, in a study of an electronic intervention, the investigators can expect a lost to follow-up of 11.0%, which may be as low as 4.4% or as high as 20.1%. Likewise, for a non-pharmacological intervention, an investigator could estimate the sample size required to attain a recruitment rate of 59.3% with a margin of error about 16.5% wide.

Many of our findings are not surprising. It is reasonable to expect challenges in recruiting people with mental health issues. Other studies have highlighted these concerns and proposed solutions in the broader population [13] and for specific co-existing conditions [14].

In principle, if the study is carefully explained to participants, few enrolled participants would withdraw from the study before randomization. While randomization was generally high, one could speculate that in studies that used incentives, participants may have viewed a 50% chance of receiving the intervention unfavorably and chose to withdraw. Run-in periods might be an effective strategy to identify participants who are likely to drop out if they are used appropriately [15]. Alternatively, investigators could identify the factors linked to pre-randomization withdrawals in the pilot trials and take measures to address them in the design of the larger trial [16].

Lower non-compliance in factorial trials, as we found, is not unexpected given that participants in factorial trials experience a higher burden especially if they are randomized to more than one active treatment [17]. It is possible that the 7 trials included in these analyses had other characteristics that may have enhanced compliance. Other studies have reported low compliance with psychotherapy interventions, albeit in fragmented population groups. For example, in one systematic review, the authors report on adherence to online psychological interventions [18]. In another, compliance is investigated only in group interventions in patients with psychosis [19]. In another systematic review, compliance is explored from the therapists' perspective for children and adolescents [20].

With regard to loss to follow-up, other studies have found that industry-funded studies may be methodologically different from others [21, 22]. This may be linked to the level of resources available and which may be deployed in this case to enhance follow-up. Educational interventions may require more engagement from participants and therefore be more inconveniencing and challenging to accommodate in their broader lives [23], leading to higher rates of loss to follow-up.

Discontinuation was low in trials from certain regions. This may have to do with local factors such as proximity to the health facility or rural dwelling, which have been shown to be linked with discontinuation [24, 25]. Discontinuation may also be high in people with HIV who also have cancer owing to the higher burden of disease, burden of treatment, and risk of death before the trial end date [26].

Studies using electronic interventions had the lowest number of people analyzed. This may be because of challenges in ascertaining participants status (given the virtual nature of the interventions) and difficulties in determining the causes of missing data. In this context, discontinuation may be related to non-usage of the electronic device precluding further meaningful participation in the trial. In skin cancer prevention research, dropout rates are higher for digital interventions than others [27].

Region-specific differences in outcomes are not uncommon in methodological research but are sometimes challenging to explain. We found high recruitment and randomization rates in studies conducted in more than one (mixed) WHO region. Larger multicenter and multi-country studies are likely to have more resources including access to methodologists and the means to ensure higher participation in trials. If conducting a trial across multiple sites or countries is indicative of study size, the literature suggests that larger studies are reported more clearly [21, 22, 28] and may have additional methodological strengths. We also found the lowest discontinuation in the South East Asia region. The implications of this finding are unclear.

There are several caveats to the use of these data. First, the availability of data was not uniform across studies and therefore not all studies contributed to all the estimates. However, we conducted a sensitivity analysis pooling data from the 62 studies that contributed data to all the outcomes and found consistent results. The second caveat is that outcomes may have been defined differently, especially in the studies that did not display a CONSORT flow diagram, and in some instances, adjudication was required to determine if participants were lost to follow-up or had discontinued. Third, our measure of non-compliance does not capture the reason for non-compliance (it could be because the intervention was not delivered appropriately, the participants did not adhere to the intervention, or there were technical and logistic issues that precluded compliance). While the result is the same, the reasons may be of value to investigators of pilot and feasibility trials. Fourth, the numbers analyzed were extracted as reported by the authors and may reflect additional approaches used to ensure complete data, including imputation techniques. Fifth, some outcomes may be influenced by time. For example, it is possible that participants are more likely to drop out from or discontinue longer studies. We invite investigators to consider this as they use these data.

## Conclusion

We have presented a large body of evidence on credible estimates for feasibility outcomes in HIV clinical trials and shown that key study characteristics may influence these estimates. These data should be used to inform the choice of thresholds for feasibility outcomes and the development of progression criteria in HIV pilot randomized trials.

## Data Availability

The datasets used and/or analyzed during the current study are available from the corresponding author on reasonable request.

## Abbreviations

CI: Confidence intervals
CONSORT: Consolidated Standards of Reporting Trials
HIV: Human immunodeficiency virus
MSM: Men who have sex with men
PLWH: People living with HIV
PWID: People who inject drugs
RCT: Randomized controlled trial
WHO: World Health Organization
